# Dermal exposure to immunostimulants induces changes in activity and proliferation of coelomocytes of *Eisenia andrei*

**DOI:** 10.1007/s00360-012-0710-7

**Published:** 2012-09-27

**Authors:** Joanna Homa, Anna Zorska, Dawid Wesolowski, Magdalena Chadzinska

**Affiliations:** Department of Evolutionary Immunology, Institute of Zoology, Jagiellonian University, Gronostajowa 9, 30-387 Krakow, Poland

**Keywords:** Coelomocytes, Immunostimulants, Proliferation, Heat shock protein 72, Nitric oxide, Respiratory burst

## Abstract

Due to the specific habitat conditions in which they live, earthworms are constantly exposed to pathogens. Consequently, they have evolved various immuno-defense mechanisms, including cellular (coelomocytes) and humoral responses, which may help to eliminate deleterious micro-organisms but also repair and/or protect host cells and tissues. Similar to mammalian phagocytes, coelomocytes can kill ingested pathogens with reactive oxygen species (ROS) and nitric oxide. In the present work, we studied the effects of the dermal exposure of *Eisenia andrei* earthworms to different immuno-stimulants: phorbol-12-myristate-13-acetate (PMA), lipopolysaccharide (LPS) or concanavalin A (ConA). After 3 days of treatment with all immuno-stimulants, decreased numbers and changed composition of the coelomocytes were observed. The immuno-stimulants also induced numerous changes in bactericidal activity, including ROS production. Furthermore, all stimulants increased cell proliferation while only LPS-treatment significantly elevated apoptosis of coelomocytes. These results demonstrate that in vivo treatment of earthworms with immuno-stimulants induces various changes in their coelomocyte response.

## Introduction

Earthworm innate immunity is maintained by immuno-competent cells—coelomocytes, localized in the coelomic cavity. Depending on the classification, they can be further divided into three different cell populations: hyaline amoebocytes, granular amoebocytes and eleocytes/chloragocytes (Adamowicz 2005; Cooper et al. 2002; Kurek et al. 2007) or five: leucocytes type I (basophilic) and II (acidophilic), neutrophils, granulocytes and eleocytes (Calisi et al. 2009). All types of coelomocytes can recognize foreign materials (e.g. pathogens) and carry out phagocytosis and encapsulation (Engelmann et al. 2004; Kalaç et al. 2002; Popović et al. 1998). Similar to vertebrates, invertebrate immuno-competent cells also possess the capacity to combat pathogens using highly reactive metabolites, such as superoxide radicals (O^2−^), hydroxyl radicals (OH^•^), hydrogen peroxide (H_2_O_2_) and nitric oxide (NO) (Rivero 2006; Valembois et al. 1994; Valembois and Lassègues 1995). In addition, chloragogen tissue derived eleocytes are responsible for maintaining the constant pH of coelomic fluid and the storage of glycogen and lipids (Affar et al. 1998; Fischer and Molnár 1992; Prentø 1979). An interesting feature of eleocytes is their ability to autofluorescence, originating mainly from riboflavin which is located in the chloragosomes of these cells (Cholewa et al. 2006; Koziol et al. 2006; Peeters-Joris 2000; Plytycz et al. 2007). Apart from cellular components, coelomic fluid contains numerous humoral immune factors, such as lysozyme, agglutinins (e.g. lectins), fetidins, lysenins and calcium-binding proteins (e.g. calreticulin) (Bilej et al. 2000; Engelmann et al. 2004; Kauschke et al. 2007; Silerova et al. 2007). Some of these humoral factors function as opsonins facilitating phagocytosis (Bilej et al. 1990; Kalaç et al. 2002) while some others, e.g. the coelomic cytolytic factor (CCF), act as pattern recognition molecules. The latter molecules bind pathogen associated molecular patterns (PAMPs), including the peptidoglycan of Gram positive bacteria but also β-1,3-glucan from yeast or lipopolysaccharide (LPS) from Gram negative bacteria, and initiate serine protease cascades activating the prophenoloxidase (pro-PO) system (Bilej et al. 2001).

In vertebrates some PAMPs can act as mitogens, i.e. molecules that induce the polyclonal overreaction of lymphocytes. In particular, LPS induces strong stimulation of B and T cells (Goodman and Sultzer 1979; Tough et al. 1997); however, it has also been shown that LPS-induced production of nitric oxide (NO) inhibits macrophage proliferation (Vadiveloo et al. 2001). Some effects on cell proliferation have also been reported for phorbol-12-myristate-13-acetate (PMA) and for lectin concanavalin A (ConA) extracted from the jack-bean, *Canavalia ensiformis*. In leukocytes, PMA enhances proliferation via a protein kinase C (PKC)-dependent mechanism; however, the proliferation of cancer cell lines is inhibited by PMA (Cao et al. 2009). Also, ConA was found to increase the sub-G1 cell cycle phase as well as cell death (Currie et al. 2007; Fortier et al. 2008). As far as we are aware, very limited in vivo studies have been performed on the effects of the above mitogens/immuno-stimulants on invertebrate immune response. Holm and co-workers (2008) showed that in sea star (*Asterias rubens* L.) injection of LPS and ConA resulted in an increase in the number of coelomocytes due to cell proliferation in the coelomic epithelium, axial organ and Tiedemann body. Moreover, in snails (*Biomphalaria glabrata*) LPS injection, as well as in vitro exposure of amebocyte-producing organ to PMA, resulted in an increase in the number of dividing hematopoietic cells (Salamat and Sullivan 2009; Sullivan et al. 2011).

It was shown previously that ConA and LPS can also enhance programmed cell death (apoptosis) (Grant et al. 1995). Thus far, clear evidence of apoptosis for earthworm coelomocytes has only been shown upon treatment with heavy metals (e.g. Reinecke and Reinecke 2004) and our group showed that heavy metals (Cu, Cd, Pb) stimulate expression of the apoptosis executor protein (caspase-3) (Homa et al. 2007). Moreover, the same heavy metals stimulate expression of heat shock proteins (HSP70 and 72). HSPs are highly conserved proteins, which are constitutively expressed in all cell types, both in invertebrates and vertebrates, where they are essential for protein folding and protection from denaturation in physiological conditions, e.g. during cell cycle and cell differentiation (Kiang and Tsokos 1998). Furthermore, HSPs can protect cells from stress-induced caspase-dependent apoptosis (Parcellier et al. 2003). In addition, it has been suggested that in mammals heat shock proteins function as proteins enhancing binding and recognition of LPS (Wallin et al. 2002).

As the effects of mitogens on the invertebrate immune response are still unclear, we aimed to investigate the impact of in vivo LPS, PMA and ConA stimulation on the number and composition of *Eisenia andrei* coelomocytes, as well as their activity (respiratory burst and NO production), apoptosis and proliferation. Moreover, we verified the expression of heat shock proteins in coelomocytes retrieved from earthworms exposed to those immuno-stimulants.

## Materials and methods

### Animals and exposure condition

Adult (clitellate) earthworms (0.41–0.83 g body weight) of *Eisenia andrei* (Sav.) were collected from the stockbreeding maintained in the Institute of Zoology of the Jagiellonian University, kept in controlled laboratory conditions (16 ± 1 °C; 12:12 LD) in commercial metal-free soil (PPUH Biovita, Poland) samples in plastic boxes for at least 2 weeks for acclimatization. The experiments were conducted by the filter paper contact method (Homa et al. 2005; OECD 1984). Vijver and co-workers (2003) showed that dermal exposure may be considered as a significant route of toxicant/stimuli uptake in earthworms (Vijver et al. 2003).

After 3 h on moist filter paper (Whatman, International Ltd, UK), each worm was washed, dried and placed individually for 3 days in 15 ml vials with filter papers, that were soaked either with water (control) (as described previously in Homa et al. 2007) or with immunostimulants: lipopolysaccharide - LPS from *Escherichia coli* 0111:B4 (Sigma-Aldrich Co., St. Louis, MO, USA) (0.5, 1, 2 and 5 mg/ml), phorbol 12-myristate 13-acetate**-**PMA (Sigma-Aldrich Co., St. Louis, MO, USA) (0.01, 0.05, 0.1, 0.5, 1, 5 and 10 μg/ml) or concanavaline A – ConA (1, 2.5, 5 and 10 μg/ml) (Sigma-Aldrich Co., St. Louis, MO, USA).

All mention immunostimulants are widely used in immune response studies, both in vertebrates and invertebrates (e.g. Cao et al. 2009; Fortier et al. 2008; Salamat and Sullivan 2009). In addition some control animals were kept in a commercial soil.

### Harvesting of coelomocytes

After 3 days of exposure to the immunostimulants the earthworms were stimulated for 1 min with a 4.5 V electric current to expel coelomic fluid with coelomocytes through the dorsal pores according to the procedure described previously (Homa et al. 2008; Roch 1979).

### Flow cytometric measurement and analysis

To determine a cell composition of coelomocytes, the coelomic fluid samples were analyzed with a FACScalibur flow cytometer (BD Biosciences). During analytical experiments, 10,000 threshold events per worm sample were collected and analyzed on the basis of their forward scatter (FSC) (for cell size) and sideward scatter (SSC) (cell complexity) properties. Fluorescence FL1-H was recorded for estimation of autofluorescence of eleocytes. Data were analyzed using WinMDI 2.9 software (Joe Trotter, http://facs.scripps.edu).

### Respiratory burst

The respiratory burst activity of coelomocytes was measured with the nitroblue tetrazolium (NBT) as described previously (Chadzinska et al. 2009). Suspension of coelomocytes, 1 × 10^6^/ml was incubated with NBT (1 mg/ml, Sigma–Aldrich Co., St. Louis, MO, USA) and after 1 h incubation, the reaction was stopped with methanol. The plates were air-dried and 120 μl of 2 N potassium hydroxide and 140 μl of dimethyl sulphoxide were added to each well. The optical density (O.D.) was recorded in an ELISA reader (Perkin Elmer Luminescence Spectrometr LS50B) at 540 nm.

### Nitric oxide release

Nitrite/nitrate production, an indicator of nitric oxide synthesis, was measured in cell culture supernatants as described previously (Chadzinska et al. 2009). Briefly, 100 μl of cell culture supernatant was added to 50 μl of 1 % (w/v) sulphanilamide in 2.5 % (v/v) phosphoric acid and 50 μl of 0.1 % (w/v) N-naphthyl-ethylenediamine in 2.5 % (v/v) phosphoric acid (all from Sigma–Aldrich Co., St. Louis, MO, USA). The O.D. reading was taken at 540 nm.

### Cell cycle analysis

In order to quantify percentage of the proliferating and apoptotic cells among free-floating coelomocytes, flow cytometry analysis was performed on the cells stained with propidium iodide where intensity of propidium iodide-derived FL-2 fluorescence is proportional to DNA content in the cells (Blacklidge and Bidwell 1993). The cell suspension was incubated with “DNA buffer”: 5.1 mg propidium iodide (Sigma Chemical Co., St. Louis, MO, USA),10 mM Tris-base (Sigma Chemical Co.) 10 mM NaCl (POCH, Gliwice, Poland),700 U/l RNase (Sigma Chemical Co.), 0.1 ml/100 ml Nonidet P-40 (Sigma Chemical Co., pH 8.0) and then analyzed by flow cytometry to assess the cell cycle DNA profile.

Coelomocytes (500 μl of 1 × 10^6^ cells/ml suspension) were mounted on slides by cytospin (5 min; 1900 rpm), fixed in 4 % paraformaldehyde, cytospines, mounted in Vectashield with DAPI (Vector Labs), and analyzed in fluorescence microscopy (Axio Imager. M2, Zeiss, Germany) with imaging system AxioCam MRm (monochrome variant).

### Immuno-blot detection of heat shock protein HSP72

To examine expression of stress proteins, dot-blot assays were performed in a 96-well plate format using a Bio-dot microfiltration manifold (Bio-Rad, Hercules, CA, USA). Lysates of tissues were prepared according to the manufacture’s protocols (Roche Diagnostic GmbH, Mannheim, Germany) as described by Homa et al. (2005). The protease inhibitor cocktail PMSF (Roche Applied Diagnostic GmbH, Mannheim, Germany) was used to prepare coelomocyte extracts. The amount of protein was determined by the BCA (Sigma–Aldrich Co., St. Louis, MO, USA) method and samples were diluted to the same concentration. For analyses, 50 μl of protein was added to each well of microfiltration apparatus. Samples microfiltration blotting was performed according to the Bio-Rad protocol on to nitrocellulose membrane (Bio-Rad, Hercules, CA, USA). The membranes were blocked for 45 min at 37 °C in a blocking buffer containing 5 % non-fat powder milk (Gostyn, Poland) in TBS (20 mM Tris–HCl, 500 mM NaCl, pH 7.5). Then membranes were incubated with monoclonal anti-HSP72 biotin conjugate antibody (Stressgen, San Diego, CA, USA) diluted 1:4,000 in TTBS (TBS with 0.05 % Tween 20) containing 1 % non-fat milk and incubated overnight at 4 °C. Next day membranes were washed at room temperature by continuous shaking in TTBS. The presence of HSP72 was immunodetected with Streptavidin–Alkaline Phosphatase SAv-AKP, BD Pharmingen, San Diego, USA) after 30 min incubation at room temperature. Then the reaction was developed by BCIP/NBT (Bio-Rad, Hercules, CA, USA). The membranes were air-dried and a densitometric analysis of protein dots was performed using of the UVISoft-UVIMap program (UVItec, Ltd.).

### Data analysis and statistics

Results are expressed as means ± standard errors (X ± SE). Significant differences between means were evaluated using one-way ANOVA with post hoc Tukey test. The level of significance was established at *p* < 0.05.

## Results

### Coelomocyte number and composition

**Fig. 1:**
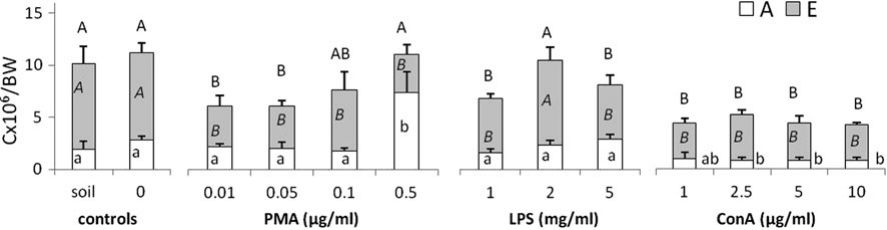
Number of coelomocytes (C) per body weight (BW) × 10^6^, amoebocytes (A) and eleocytes (E), retrieved from earthworms (*Eisenia andrei)* kept in soil (*n* = 18) or exposed to filter paper soaked either with distilled water (0 mg/ml, *n* = 18) or PMA, LPS or ConA (*n* = 9–12). X + SE. Different letters indicate the values that are significantly different according to ANOVA (e.g. “a” and “b”) while the values sharing the same letter (e.g. “a” and “ab”, “a” and “a”) are similar i.e. not statistically different. Capital letters (A or B) indicate differences in total number of coelomocytes between groups, capital italic letters (*A* or *B*) in number of eleocytes, while small letters (a or b) in the number of amoebocytes

**Fig. 2:**
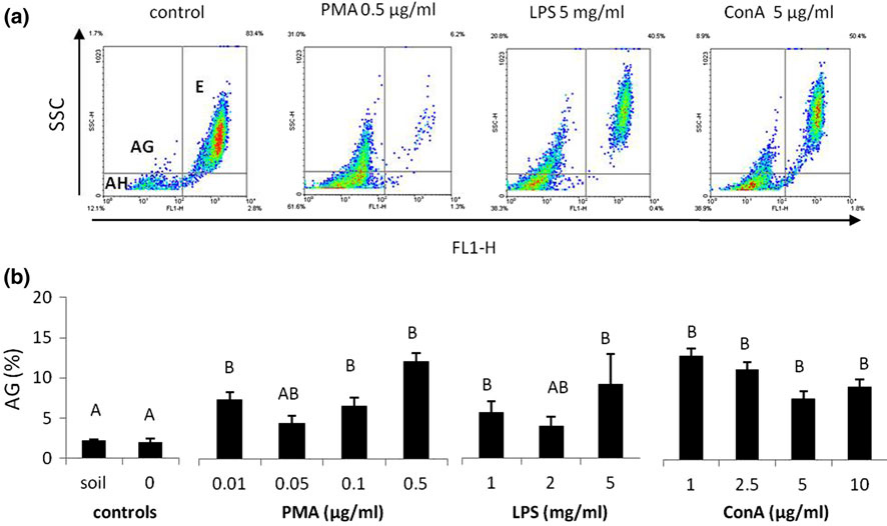
Flow cytometric analysis of coelomocytes derived from earthworms (*Eisenia andrei)* kept in soil (*n* = 18) or exposed to filter paper soaked either with distilled water (0 mg/ml, *n* = 18) or PMA, LPS or ConA (*n* = 9–12). **a** Representative density plots (FL1-H autofluorescence versus cell complexity SSC-H) of coelomocytes from worms exposed to filter paper soaked with distilled water (control) or PMA (0.5 μg/ml), LPS (5 mg/ml) or ConA (5 μg/ml). Gates set on the density plot indicate three populations of coelomocytes: hialine amoebocytes (AH), granular amoebocytes (AG), and eleocytes (E). **b** Percentage of granular amoebocytes (AG). X + SE. Different letters indicate the values that are significantly different according to ANOVA (e.g. “A” and “B”) while the values sharing the same letter (e.g. “A” and “AB”, “A” and “A”) are similar i.e. not statistically different

The number of cells obtained from animals kept on the filter paper soaked with PMA (0.01 and 0.05 μg/ml), LPS (1 and 5 mg/ml) or ConA (1–10 μg/ml) was lower compared to the control groups (soil and H_2_O). In animals treated with PMA and LPS cell reduction was particularly visible in the case of eleocytes, while ConA decreased numbers of both amoebocytes and eleocytes (Fig. 1). The highest concentration of PMA (0.5 μg/ml) did not change total coelomocyte numbers, but induced changes in cell composition, i.e. it increased the number of amoebocytes and reduced the number of eleocytes (Fig. 1). The changes observed under the microscope were additionally confirmed by flow cytometric analyses. These revealed that LPS, ConA, and especially PMA-treatment, significantly reduced the numbers of highly granular and autofluorescent cells (Fig. 2a). Moreover, flow cytometry analysis showed changes in the amoebocyte composition, as all stimulants increased in terms of the percentage of granular amoebocytes (AG), but not of hyaline amoebocytes (AH) (Fig. 2ab).

### Production of reactive oxygen species and nitric oxide

**Fig. 3:**
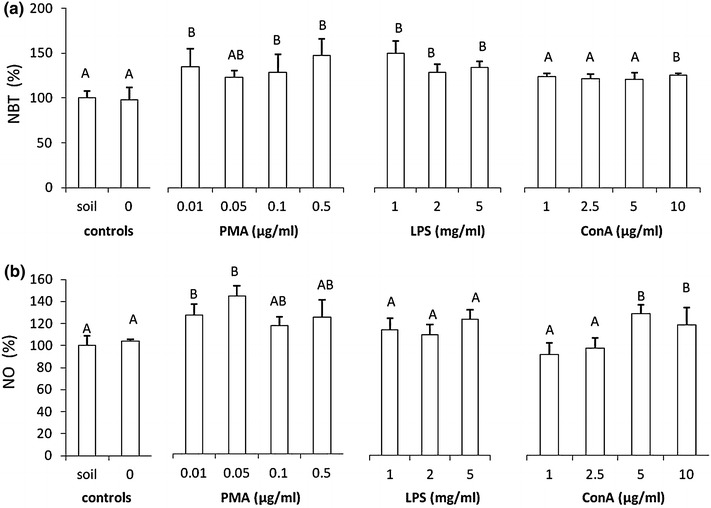
Production/release of reactive oxygen species and nitric oxide (NO) in coelomocytes derived from earthworms (*Eisenia andrei)* kept in soil or exposed to filter paper soaked either with distilled water (0 mg/ml) or PMA, LPS or ConA. **a** Nitroblue tetrazolium (NBT) reduction. **b** NO level. X + SE, *n* = 6. Different letters indicate the values that are significantly different according to ANOVA (e.g. “A” and “B”) while the values sharing the same letter (e.g. “A” and “AB”, “A” and “A”) are similar i.e. not statistically different

NBT reduction significantly increased production of reactive oxygen species and nitric oxide in coelomocytes derived from animals treated with PMA (0.01, 0.1 and 0.5 μg/ml) and LPS (1–5 mg/ml), whereas in animals treated with ConA only the highest concentration (10 μg/ml) significantly increased the NBT reduction (Fig. 3a).

Also, the production/release of NO increased in coeolomocytes retrieved from animals treated with PMA (0.01 and 0.05 μg/ml) and ConA (5 and 10 μg/ml); however, LPS did not induce significant changes in the NO level (Fig. 3b).

### Coelomocyte proliferation and apoptosis

**Fig. 4:**
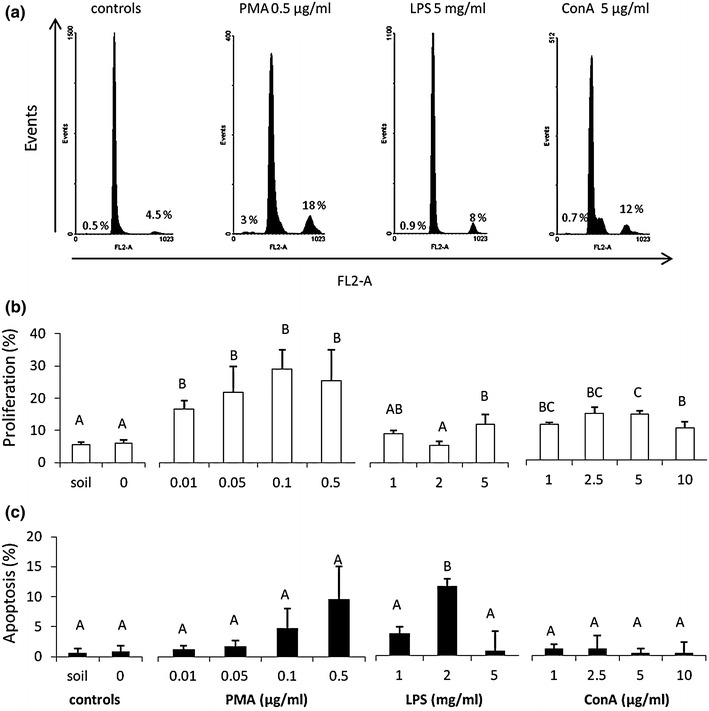
Flow cytometric analysis of the percentage of proliferating and apoptotic coelomocytes derived from earthworms (*Eisenia andrei)* kept in soil (*n* = 18) or exposed to filter paper soaked either with distilled water (0 mg/ml, *n* = 18) or PMA, LPS or ConA (*n* = 9–12). **a** Representative DNA histograms of coelomocytes from worms exposed to filter paper soaked with distilled water (control) or PMA (0.5 μg/ml), LPS (5 mg/ml) or ConA (5 μg/ml). **b** Percentages of proliferating coelomocytes **c** Percentages of apoptotic coelomocytes. X + SE. Different letters indicate the values that are significantly different according to ANOVA (e.g. “A” and “B”) while the values sharing the same letter (e.g. “A” and “AB”, “A” and “A”) are similar i.e. not statistically different

The percentage of proliferating coelomocytes was comparable in the case of control animals, i.e. the earthworms obtained from soil, and those kept for 3 days on water-soaked filters (approximately 5 %), while both PMA and ConA increased the percentage of proliferating cells in the coelomic cavity to approximately 20 and 10 %, respectively. Only the highest concentration of LPS (5 mg/ml) induced significant coelomocyte proliferation (Fig. 4a, b). Moreover, LPS in a concentration of 2 mg/ml increased coelomocyte apoptosis. In animals treated for 3 days with PMA a tendency to increased coelomocyte apoptosis was observed, while ConA did not affect coelomocyte apoptosis at the concentrations employed (Fig. 4c).

**Fig. 5:**
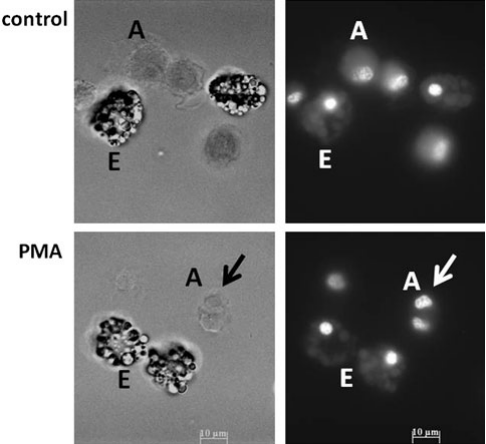
Representative microphotographs of *Eisenia andrei* DAPI-stained coelomocytes (amoebocytes - A, eleocytes - E) retrieved from animals exposed to filter paper soaked with distilled water (control) or PMA (0.01 μg/ml). Bright-field (left panel) and corresponding fluorescence microphotographs (*right panel*). *Arrow* indicates cell division

Coelomocyte DAPI staining revealed that some of the amoebocytes, but not the eleocytes, retrieved from immuno-stimulant-treated animals showed clear division (mitotic morphology) (Fig. 5).

### Expression of heat shock proteins HSP72

**Fig. 6:**
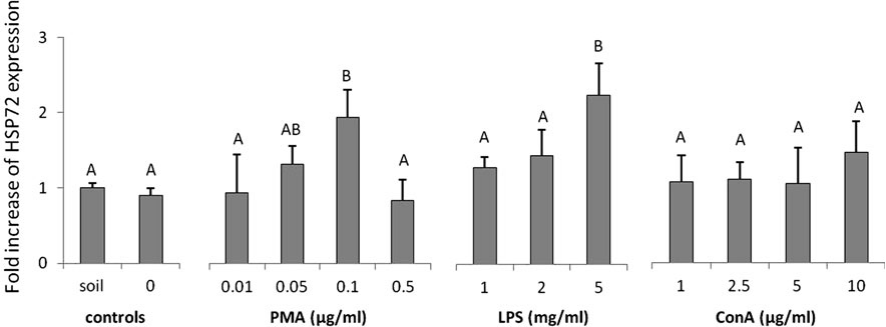
Fold increase of heat shock protein (HSP72) expression (measured densitometrically) in coelomocyte lysates derived from earthworms (*Eisenia andrei*) kept in soil (n = 18) or exposed to filter paper soaked either with distilled water (0 mg/ml, *n* = 18) or PMA, LPS or ConA (*n* = 9–12). X + SE. Different letters indicate the values that are significantly different according to ANOVA (e.g. “A” and “B”) while the values sharing the same letter (e.g. “A” and “AB”, “A” and “A”) are similar i.e. not statistically different

Both PMA (0.5 μg/ml) and LPS (5 mg/ml) treatments, but not ConA, induced significant up-regulation of HSP72 expression in earthworm coelomocytes (Fig. 6).

## Discussion

The internal defense mechanisms of annelids involve phagocytosis, nodule formation, encapsulation, blood coagulation and wound repair (Cooper 1996; Valembois et al. 1992). All these processes as well as reactions to toxicants (e.g. heavy metals) lead to a reduction in the effector cell population which is followed by the reconstruction of their resources (Homa et al. 2007, 2008). In the present study, we revealed that in vivo dermal exposition of the earthworm *Eisenia andrei* to different immuno-stimulants induces changes in the number, composition and activity of coelomocytes. Moreover, we found that a reduction in the total number of coelomocytes increased cell proliferation. The most potent compounds causing this process are PMA and ConA, while LPS induces much weaker cell proliferation.

Currently, we cannot clearly state whether the reduction in the number of cells in the coelom results from their spontaneous release after dermal irritation with immuno-stimulants or whether it is related to aggregation and/or formation of brown bodies inside the cavity. Valembois and co-workers (1992) demonstrated that in earthworms the formation of brown bodies and their melanisation in the coelomic cavity may result from aggregation of coelomocytes around foreign cells, such as bacteria and gregarines (Valembois et al. 1992). Thus, it might be that also in the present experiments the activated coelomocytes were “arrested” in capsules/aggregates and could not be retrieved by electric shock stimulation. This hypothesis may be supported by the observation that after 48 h of in vitro incubation with mitogens (especially ConA) both amoebocytes and eleocytes can form aggregates (data not shown).

Interestingly, dermal exposure of earthworms to all stimulants resulted in changes in cell composition. In the case of animals treated with stimulants, a reduction in the percentage of elocytes and an increase in the percentage of granular amoebocyes was observed. Based on the observations of cell morphology and analysis of their autofluorescence (FL-1 fluorescence) we can exclude the possibility that cells described here as granular amebocytes are partially degranulated eleocytes. However, we cannot exclude the possibility that the observed increase in amobeocyte granularity, demonstrated by the increase in side scatter (SSC), results from the cell activation and elevation of their lysosome numbers. A similar effect was observed in the case of human peripheral blood mononuclear cells (PBMC) after PMA treatment (Opper et al. 2010). It was also shown that bacterial infection of earthworms *Eisenia fetida* increased the numbers of lysosomes in coelomocytes (Engelmann et al. 2004). The increased number of granular amebocytes might alternatively result from their enhanced proliferation. In the earlier experiments, we showed that experimental ceolomocyte depletion from *Dendrobaena veneta* stimulates proliferation of coelomocytes (Homa et al. 2008). In the present experiments, amebocytes, consist the main cell-proliferating population in the coelom of immuno-stimulant-treated earthworms. Moreover, our previous experiments revealed that proliferation of eleocytes/chloragocytes can be found mainly in the typhlosole (Olchawa et al. 2006) suggesting that the main population of cells proliferating in the coelomic cavity are amebocytes. In addition, in vitro tests showed that ConA, LPS or PHA stimulate proliferation of *E. fetida* coelomocytes (Cooper et al. 1995; Roch et al. 1975).

Furthermore, the data presented here suggest that the elevated contribution of the granular amebocytes to the pool of coelomic cells influences the killing activity of the cells as measured by the respiratory burst and the release of nitric oxide. In vertebrates, it is well known that immuno-stimulants, such as LPS and PMA induce production of proinflammatory mediators such as NO and ROS, respectively (O’Neill 2011; Yamada et al. 2006). Our results also demonstrate that PMA and LPS stimulated ROS generation in *E. andrei* coelomocytes. Previously, differences in ROS generation upon treatment with different immuno-stimulants have been observed in hemocytes of the American lobster (*Homarus americanus*) stimulated *inter alia* with ConA, LPS or PMA (Anderson and Beaven 2005). In this study, exposure to PMA induced a strong ROS response, while ConA and LPS failed to stimulate O2- generation (Anderson and Beaven 2005). In the case of earthworm coelomocytes, elevation of the respiratory burst was observed in vitro upon zymosan stimulation (Valembois and Lassègues 1995) and in vivo during brown body formation (Valembois et al. 1994). Several reports have described NO synthesis/release by invertebrate hemocytes originating from e.g. echinodermates (Beck et al. 2001), insects (Nappi et al. 2000) and snails (Wright et al. 2006), thus indicating that NO is the evolutionally conserved product of phagocytes (Beck et al. 2001). In our experiments, PMA and ConA, but surprisingly not LPS, stimulated production/release of NO by coelomocytes, while production of ROS was up-regulated in cells from animals treated with different doses of LPS and PMA. On the other hand, a significantly elevated percentage of apoptotic cells was observed only in coelomocytes retrieved from animals treated with LPS (2 mg/ml). One should keep in mind that production of reactive oxygen species, viewed as one of the first defense lines against invading pathogens, might also damage host cells, e.g. by inducing cell apoptosis. Therefore, the increased ratio of apoptotic coelomocytes upon LPS treatment could explain the simultaneously decreased NO release and weak cell proliferation.

Furthermore, elevated production of reactive oxygen species might stimulate a protective HSP response (Jacquier-Sarlin et al. 1994), which subsequently can interfere with the apoptotic pathway stimulation and protect cells from the stress-induced caspase-dependent apoptosis (Parcellier et al. 2003). For this expression of HSPs, expression such as the HSP72 protein is a good marker of cell stress (Kiang and Tsokos 1998). In our earlier experiments, we showed that exposure to heavy metals and high temperature induces expression of the HSP72 protein in coelomocytes (Homa et al. 2005, 2007; Kurek et al. 2001). In the present work, an increased level of heat shock proteins was observed in coelomocytes retrieved from animals treated with high concentrations of PMA or LPS, but not ConA. A similar increase in HSP72 expression was observed in U937 monocytes differentiated to macrophage-like cells upon PMA treatment (Twomey et al. 1993), while in lymphocytes HSP expression was induced with ConA (Ghassemi et al. 1991). However, in turn the increase in HSP levels in macrophages inhibited the release of cytokines, oxygen free radicals or nitric oxide and reduced the bactericidal capacity of the organism (Kiang and Tsokos 1998, Mochida et al. 2007). In the present work, HSP72 levels did not correlate with the synthesis of ROS and NO, while the potential anti-apoptotic role of HSP72 could only be seen in the case of earthworms treated with 5 mg/ml of LPS. At this concentration, the expression of HSP72 was significantly higher, while the percentage of apoptotic cells was low.

It should be stated here that mammalian leukocytes, including phagocytes, can be activated in vitro with very low LPS concentrations, down to 10 pg/ml (e.g. Mészáros et al. 1994), while cold-blooded vertebrates and invertebrates are much less sensitive to LPS. For example, insect hemocytes show significant in vitro response only when treated with 1–10 mg/ml of LPS (Wittwer et al. 1997). Consequently, in the current study we used concentrations of LPS and PMA that were higher in comparison to those used in vertebrates, while in the case of ConA we based our approach on earlier in vitro experiments (Cooper et al. 1995). The lower sensitivity of earthworms to LPS may be connected with their role *inter alia* in organic matter degradation and their continuous contact exposure to soil bacteria and other micro-organisms. The natural behavior of earthworms is increasingly being utilized for vermicomposting, and the extent to which earthworms promote the survival and dispersal of the bacterium (Williams et al. 2006).

In the present work we showed for the first time that dermal exposure of earthworms to various immuno-stimulants causes a broad range of changes in the number and activity of coelomocytes, including alternations in the production of oxygen free radicals and nitric oxide, and the expression of heat shock proteins, as well as cell proliferation and apoptosis. Furthermore, we demonstrated a different sensitivity of earthworms/earthworm coelomocytes to various stimulants. However, further work aimed to explain mechanisms of these processes is still required.
